# Systematic review: Impact of stem cells-based therapy, and platelet-rich plasma in hair loss and telogen effluvium related to COVID-19

**DOI:** 10.1016/j.reth.2023.07.001

**Published:** 2023-07-21

**Authors:** Pietro Gentile, Simone Garcovich

**Affiliations:** a Plastic and Reconstructive Surgery, Department of Surgical Science, "Tor Vergata" University, Rome, 00133, Italy; b Academy of International Regenerative Medicine & Surgery Societies (AIRMESS), 1201 Geneva, Switzerland; c Università Cattolica del Sacro Cuore, 00168, Rome, Italy

**Keywords:** COVID-19 and hair loss, COVID-19 and telogen effluvium, SARS-CoV-2 and hair loss, Regenerative plastic surgery, PRP in COVID-19, Plastic surgery

## Abstract

**Background:**

The incidence of hair loss (HL) and telogen effluvium (TE) in COVID-19 patients has been reported in several studies.

**Objectives:**

Evaluate both the increased incidence of HL and TE in COVID-19 and the effectiveness of Platelet-Rich Plasma (PRP), Adipose-derived Mesenchymal Stem Cells (AD-MSCs), and Human Follicle Stem Cells (HFSCs) in these patients.

**Methods:**

The protocol was developed by the Preferred Reporting for Items for Systematic Reviews and Meta-Analyses-Protocols (PRISMA-P) guidelines. A multistep search of PubMed, MEDLINE, Embase, Clinicaltrials. gov, Scopus, and Cochrane databases has been performed to identify papers focusing on HL and TE COVID-19 related, and papers focusing on AD-MSCs, HFSC, and PRP use.

**Results:**

Of the 404 articles initially identified focusing on HL and TE, 44 were related to COVID-19, and finally, only 6 were analyzed. On the other way, 331 articles focusing on AD-MSCs, HFSC, and PRP were initially identified. Of these, only 6 articles PRP (n = 3), AD-MSCs, and HFSCs (n = 3) have been analyzed.

**Conclusion:**

Collected data confirmed both an increased incidence of HL and TE in COVID-19 patients, preliminarily, the related effectiveness of AD-MSCs, HFSCs, and PRP without major side effects.

## 1 Introduction

Several types of baldness and hair loss (HL) have been described in the literature, of these most commonly, male- or female-pattern hair loss (MPHL or FPHL), androgenetic alopecia (AGA), alopecia areata (AA), and telogen effluvium (TE). As known, the cause of MPHL is a combination of genetics and male hormones; the cause of FPHL is yet unclear; the cause of AA is autoimmune, and the cause of TE is typically a physically or psychologically stressful event [[1], [2], [3], [4]]. In AGA, the most common disorder, miniaturization of the follicles has resulted in a diminishment of the anagen phase, with an improvement in the number of resting hair follicles, telogen, containing microscopic hairs in a hairless scalp [[5], [6], [7]]. In baldness, hair follicle stem cell numbers stay unaltered, though the number of more actively proliferating progenitor cells particularly diminishes [8]. Starting in 2020, an evolving body of literature has associated coronavirus disease 2019 (COVID-19) with primary mucosal, hair, nail, and skin complaints, which may precede the classic symptoms of COVID-19 in some cases. Pruritic erythematous rash and/or patchy exanthemata's red rash on the trunk appears to be the most common cutaneous findings. Acro ischemic lesions or “COVID toe”, which are micro thrombotic presentations of COVID-19, may occur in both children and adults [9,10]. HL disorders have also been an important area of concern during the COVID-19 pandemic. A web-based evaluation of public dermatologic interests using Google Trends revealed that ‘hair losses were among the most searched dermatology-related terms in several European countries [11]. A simultaneous rise in public apprehension about HL along with the rising number of COVID-19 cases has been indicative of a connection. The present systematic review attempts to clarify these pathophysiological connections and discuss a novel stem cell approach based on Adipose-derived Mesenchymal Stem Cells (AD-MSCs), Human Follicle Stem Cells (HFSCs), Platelet-Rich Plasma (PRP), for the treatment of HL in individuals suffering from COVID-19 or its psychological consequences.

## 2 Methods

### 2.1 Study overview

This study has been the object of a research contract between the author P.G. and the “Tor Vergata” University, approved by Rectoral Decree R. D n. #1467/2017, continued in associate professor contract #13489/2021, and of a research project approved and supported by the University “Tor Vergata” called “*Evaluation of the potential use of regenerative strategies (Platelet Rich Plasma and Adipose-derived Mesenchymal Stem Cells) in the treatment of diseases associated with COVID-19 (Alopecia and cutaneous and subcutaneous deficiency)*” with Unique Project Code (CUP): **E83C22001960005.**

### 2.2 Search strategy and selection criteria

**Scheme 1:**
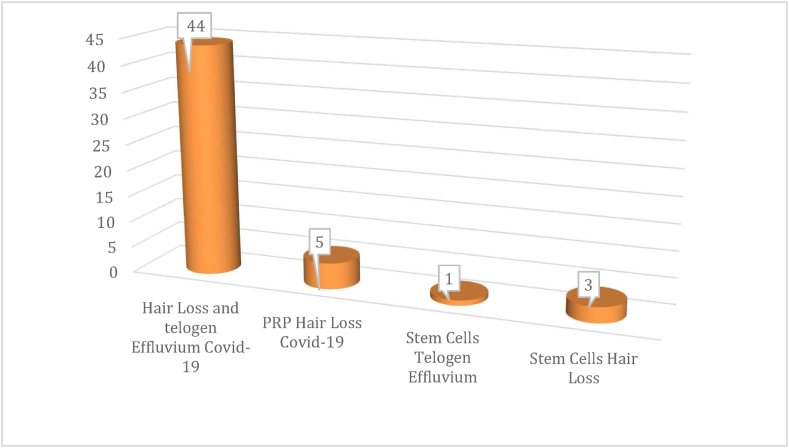
Papers were initially found on Stem Cells, and PRP applications in hair loss and telogen effluvium related to COVID-19.

This systematic review has been conducted by two investigators (P.G. and S.G.) by the Preferred Reporting Items for Systematic Reviews (PRISMA) (http://www.prisma-statement.org) [12] and the Cochrane Handbook [13]. A multistep search of the PubMed, MEDLINE, Embase, Pre-MEDLINE, Ebase, Clinicaltrials.gov, Scopus, and Cochrane databases was performed to identify studies, published before April 1, 2022, relating to the correlation between TE, HL disorders, and severe acute respiratory syndrome coronavirus 2 (SARS-CoV-2) inducing COVID-19, analyzing respectively the biomolecular way of SARS-CoV-2 in HL and the potential role of regenerative strategies represented by AD-MSCs, HFSCs, and PRP searching without a language or publishing-time restriction. Original articles including *in vivo*, *ex vivo*, and *in vitro studies,* were all eligible for inclusion. Exclusion criteria were unpublished investigations, conference reports, and lack of raw data. 404 articles using the keyword *“hair loss and telogen effluvium*” were initially found, of which only 44 articles were related to “*hair loss and telogen effluvium COVID-19*”. A total of 562 articles using the keyword *“regenerative hair loss”* was found*,* where 69 articles were related to *“Adipose stem cells hair loss”,* 50 articles to *“Follicle stem cells hair loss”,* and 212 articles to *“PRP hair loss”.* Of these, only 5 articles using the keyword *“PRP hair loss COVID-19”,* 1 article using the keyword *“Stem cells telogen effluvium COVID-19”, and* 3 articles using the keyword *“Stem cells hair loss COVID-19”* were found, as reported in Scheme 1.

### 2.3 Study assessment

This paper has aimed to assess the selected articles comparing the application of AD-MSCs, HF-MSCs, and PRP to any control for HL, and TE-related to COVID-19. Articles included in this work had to match predetermined criteria according to the PICOS (patients, intervention, comparator, outcomes, and study design) approach (https://ro.ecu.edu.au/cgi/viewcontent.cgi?referer=https://www.google.it/&amp;httpsredir=1&amp;article=1010&amp;context=ecupres). Study assessment was based on inclusion and exclusion criteria (Table 1).

**Table 1:** Study assessment based on inclusion and exclusion criteria according to the PICOS (patients, intervention, comparator, outcomes, and study design) approach (https://ro.ecu.edu.au/cgi/viewcontent.cgi?referer=https://www.google.it/&amp;httpsredir=1&amp;article=1010&amp;context=ecupres).

	Inclusion Criteria
**P**-Patients	age 18–80 years, patients with HL disorder related to COVID-19
**I**-Intervention	Stem cell-based therapy, AD-MSCs, HFSCs, and PRP
**C**-Comparator	Any type of control, internal, external, and different procedures
**O**-Outcomes	Correlation between HL disorder (including TE) and COVID-19, the impact of stem cell based-therapy, AD-MSCs, HFSC, and/or PRP in HL and TE-related to COIVD-19
**S**-Study Design	A clinical trial, randomized clinical trial, case series, case report, case-controlled studies, *in vivo, ex vivo*, and *in vitro* studies.
	***Exclusion Criteria***
**P**-Patients	HL disorder not related to COVID-19
**I**-Intervention	Minoxidil, Finasteride, steroid injections, surgical and/or other procedures
**C**-Comparator	Not applied
**O**-Outcomes	Not applied
**S**-Study Design	Expert opinion, comments, letter to the editor, articles identified as bias - not correct match with the keywords used and with the treatments -, shorter follow-up than 1 month, review, and systematic review. No limitations were applied to ethnicity.

This systemic review, performed on the PICOS approach is considered an “*Evidence-Based Medicine (EBM) 1a level study”* according to the Oxford Centre for Evidence-Based Medicine (OCEBM), March 2009 (https://www.cebm.net/2009/06/oxford-centre-evidence-based-medicine-levels-evidence-march-2009/).

### 2.4 Data extraction

Data were independently extracted by the first investigator (P.G.) and checked by the last investigator (S.G.) only from the retrieved articles. Any disagreement on the extracted data has been settled by a consensus among P.G. and S.G. The following data have been extracted: first author, year of publication, study design, number of patients, type of procedure, and primary and secondary outcomes. The quality of the included investigations was independently assessed using two investigators (P.G. and S.G.) using the Cochrane Collaboration's Risk of Bias Assessment tool for randomized controlled trials (RCTs) [13] while using the Newcastle–Ottawa Scale to evaluate the individual non-randomized studies [14].

### 2.5 Endpoint definition

The primary endpoint was to clarify the pathophysiological connections between HL, TE, and COVID-19 showing an increasing incidence, while the secondary endpoint, was to discuss a novel stem cell-based approach analyzing the impact of AD-MSCs, HFSCs, and PRP, for the treatment of HL in individuals suffering from COVID-19.

## 3 Results

### 3.1 Study selection

**Scheme 2:**
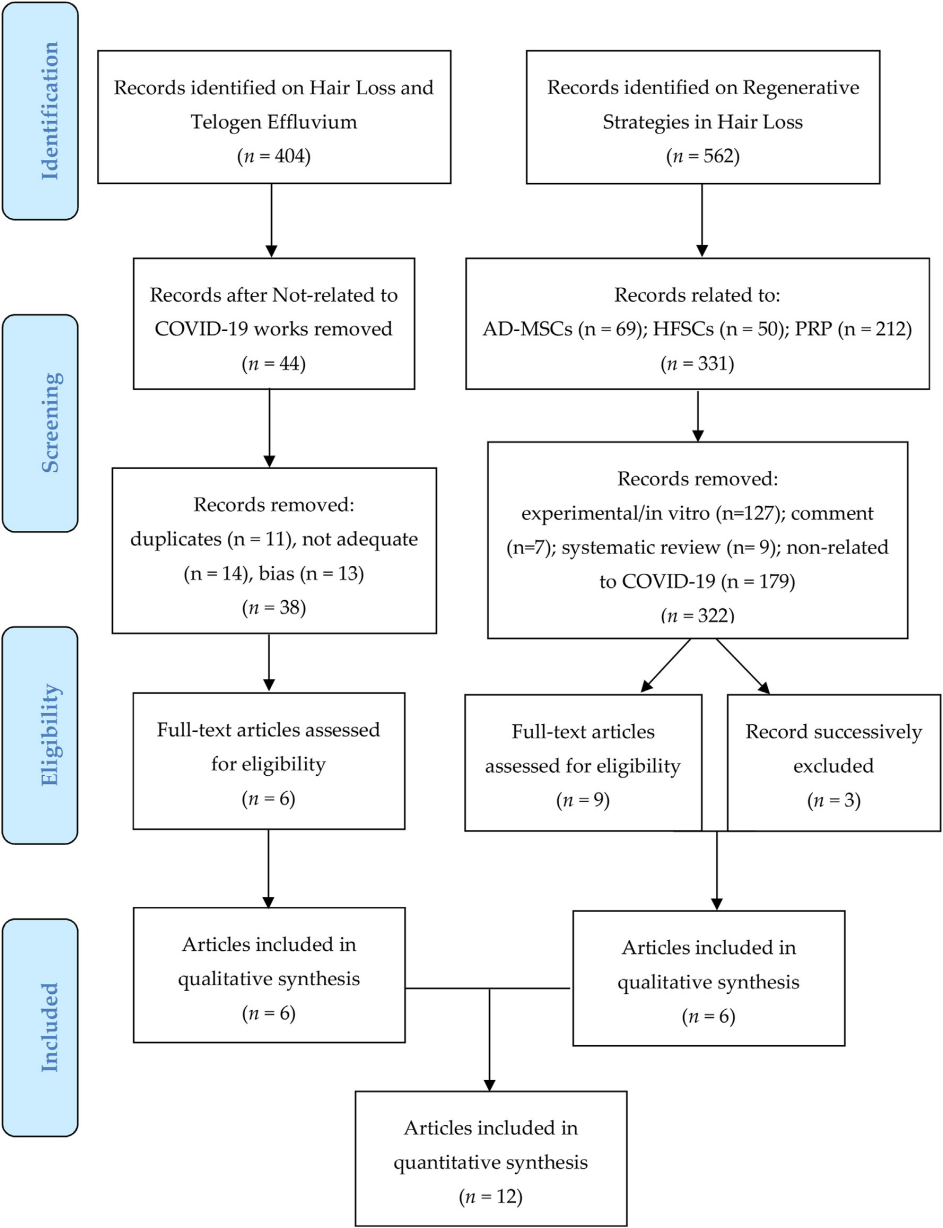
PRISMA Flow (Preferred reporting items for systematic review and meta-analysis).

404 articles focused on HL and TE were initially identified and selected using Prisma Flow (www.prisma-statement.org) (Scheme 2). A total of 360 articles were excluded due to not being related to COVID-19. 44 articles were selected. Of this amount, 11 were duplicates, 14 were not adequate (review, letter to the editor, comment on, short communication), and 13 articles were considered biased (not correctly matched with the treatment and keywords used). Consequently, only 6 articles regarding HL and TE related to COVID-19 were analyzed.

562 articles focusing on stem cell-based therapy and regenerative strategies in HL were initially identified. Of this amount, 331 articles related to AD-MSCs (n = 69), HFSC (n = 50), and PRP (n = 212) were selected. Of these, 127 articles were identified as experimental, 16 articles identified as a comment (n = 7), a systematic review (n = 9), and 179 articles not focused on COVID-19 were excluded. Only 9 original articles strictly and exclusively focused on the use of PRP (n = 5), AD-MSCs, and HFSCs (n = 4) in HL and TE-related to COVID-19 were analyzed. Of these, 3 articles have been successively removed (2 articles on PRP as biased, and 1 article on stem cells as duplicate). Finally, 6 articles have been analyzed.

Consequently, a total of 12 articles (n = 6 regarding HL and TE-related to COVID-19, n = 3 regarding PRP use in HL COVID-19 related, n = 3 regarding Stem Cell use in HL COVID-19 related) were included and analyzed in this systematic review as showed in Prisma Flow (Scheme 2).

### 3.2 Literature analysis and study subjects reporting hair loss increasing in COVID-19 patients

6 articles were included in the quantitative synthesis. Clinical surveys conducted in Spain [15,16] and India [18] demonstrated higher rates and severity of pattern hair loss (PHL) in hospitalized COVID-19 patients compared to age-matched, non-infected populations. The first preliminary study that became available was a descriptive study on 41 Caucasian males admitted to hospitals in Spain with a diagnosis of bilateral SARS-CoV-2 pneumonia (mean age = 58 years). 71% of the individuals were diagnosed with significant MPHL of which 39% had a severe involvement [15]. A follow-up, multicenter study on 175 confirmed COVID-19 patients verified those preliminary findings and reported that 79% of men and 42% of women had significant PHL. These values are in sharp contrast with the expected prevalence rates among age- and race-matched populations. The prevalence of MPHL in a similar white population is expected to be at 31–53%, and that of FPHL to be at a maximum of 38% [16,17]. Hence, the data available up to date points to a considerably higher prevalence and severity of PHL among hospitalized COVID-19 patients. Notably, patients with higher stages of HL had worse clinical outcomes (use of ventilators and deaths). Some authors proposed the eponym ‘*Gabrin sign’* to refer to the phenomenon of severe baldness in COVID-19 patients with a higher risk of unfavorable outcomes [19]. To elucidate whether this correlation is driven by a causal relationship or not, larger, controlled surveys in both inpatient and outpatient settings are incontrovertibly required. Beyond having a positive correlation with the SARS-CoV-2 infection, diffuse alopecia appears to be an important sequel of COVID-19. A large longitudinal study on 538 COVID-19 survivors and 184 controls was carried out in Wuhan, China, to investigate the prevalence and predictors of COVID-19 clinical sequelae [19]. Three to four months after discharge, alopecia was among the most prevalent complaints in convalescent COVID-19 patients, reported more commonly by women. Almost half of the female participants started experiencing HL after being infected by SARS-CoV-2 compared to no case in the control group. 27% of affected cases experienced HL during their hospitalization while 73% first recognized it after being discharged [19]. Due to the timing of symptoms, at least a proportion of the cases with newly-onset alopecia in this study are suspected to have premature or exacerbated FPHL. An explanation of the observed relationship between PHL and COVID-19, a key role for systemic inflammation as a common underlying pathology is conceivable. This important factor may as well justify higher grades of HL in patients with severe COVID-19 as reported by Wambier et al. [18] Hypoxemia leading to skin ischemia is another potential pathogenetic factor that connects lung damage secondary to SARS-CoV-2 infection with hair growth impairment. *Ex-vivo* and *in-vivo* experiments by Kato et al. [20] demonstrated significant reductions in hair growth rate, hair shaft size, and pigmentation in anagen hairs under ischemia [20]. The analyzed studies have been summarized in Table 2.

**Table 2:** Clinical studies on the relationship between hair loss and COVID-19 and ischemia conditions.

Authors	Specs	Outcomes
Kato H et al. [20] 2020	A pre-clinical investigation (performed in mice) reporting the effects of ischemia and hyperoxygenation on hair growth	An *ex-vivo* and *in-vivo* study showed significant reductions in hair growth rate, hair shaft size, and pigmentation in anagen hairs under ischemia.
Xiong Q et al. [19] 2020	A single-center longitudinal study on 538 COVID-19 patients in Wuhan.	Fifty percent of the female patients started experiencing HL after being infected by SARS-CoV-2. Twenty-seven percent of these patients experienced HL during their hospitalization while seventy-three percent showed HL after being discharged. HL was among the most prevalent complaints in convalescent COVID-19 patients three to four months after discharge, reported more commonly by women. Some authors proposed the eponym ‘Gabrin sign’ to refer to severe baldness in COVID-19 patients with a higher risk of unfavorable outcomes.
Wambier CG et al. [18] 2020	Investigation on AGA-related COVID-19 through age-matched epidemiologic studies and hospital outcomes analysis with or without the “Gabrin sign”.	A pathogenetic factor that connects lung damage secondary to SARS-CoV-2 infection with hair growth impairment was hypoxemia leading to skin ischemia.
Wambier CG [17]. 2020	Reply to "Comment on AGA present in the majority of patients hospitalized with COVID-19″	The data analyzed showed a considerably higher prevalence and severity of hair loss among COVID-19 patients during hospitalization.
Wambier CG et al. [16] 2020	Multi-center study on 175 patients hospitalized with COVID-19	Seventy-nine percent of males and forty-two percent of females had significant pattern hair loss in contrast with the expected prevalence rates among age- and race-matched populations.
Goren A et al. [15] 2020	A descriptive study on 41 Caucasian males hospitalized with bilateral SARS-CoV-2 pneumonia.	Seventy-one percent of the individuals have been diagnosed with MPHL of which thirty-nine percent had severe clinical COVID-19 outcomes.

### 3.3 Literature analysis and study subjects reporting the effectiveness of AD-MSCs, HFSCs, and PRP in HL COVID-19 related

6 articles were included in the quantitative synthesis (meta-analysis). Of this amount, 3 articles [[21], [22], [23]] regarding the role of stem cells in HL related to COVID-19 have been found while 3 articles [[24], [25], [26]] were related to the use of PRP. Hawwam SA et al. [21] evaluated autologous micrografts from scalp tissues as a therapeutic modality in the management of TE caused by COVID-19. 20 patients with previous COVID-19 infections suffered from TE were included in this study for HFSCs micrograft scalp treatment and they were evaluated after 3 months of treatment and after 6 months. The outcomes analyzed reported a significant improvement in hair thickness and density compared with the start of the treatment and 6 months of follow-up. Autologous micrografts based on HFSCs of the scalp showed marked improvement in the treatment of COVID-19 TE [21]. Jie MA et al. [22] described the impact of COVID-19 on skin and HL affirming that pathological features of skin tissues from patients infected with SARS-CoV-2 at a molecular level are limited. Especially, the ability of SARS-CoV-2 to infect skin cells and impact their function is not well understood. A proteome map of COVID-19 skin is established here and the susceptibility of human-induced pluripotent stem cell (hiPSC)-derived skin organoids with hair follicles and the nervous system is investigated, to SARS-CoV-2 infection. It is shown that KRT17+ hair follicles can be infected by SARS-CoV-2 and are associated with the impaired development of hair follicles and epidermis [22]. Minjin Jeong et al. [23] reported a series of ten COVID-19 patients with audiovestibular symptoms such as hearing loss, vestibular dysfunction, and tinnitus. To investigate the causal relationship between SARS-CoV-2 and audiovestibular dysfunction, they examined human inner ear tissue, human inner ear *in vitro* cellular models, and mouse inner ear tissue. Apparently, this paper is out of the main interest of this paper, but interestingly, the inner ear organoids show that hair cells express ACE2 and are targets for SARS-CoV-2, opening a new window of understanding in the relationship between HL and SARS-CoV-2 [23]. Oscar Adrian Vazquez et al. [24] described the use of Advanced platelet-rich fibrin (aPRF) reduced to an injectable form with micronization, to treat AA in a 28-year-old patient who developed it after symptomatic COVID-19 infection. The patient was vaccinated in between infections, and symptoms were limited to headache and sore throat. They reported a complete resolution of AA at a 6-month follow-up with only two treatments as opposed to monthly intralesional steroids. İşlek A et al. [25] aimed to examine prospectively the accelerated pattern of HL in nine patients with PCR positive for COVID-19 and related management with PRP treatment. Patients underwent PRP injections for four sessions. Results were accessed with the hair pull test (HPT) and self-administered hair growth questionnaire (HGQ). Nine patients were admitted with complaints of HL after an average of 220 ± 24.2 days after recovery from COVID-19. The mean age of the patients was 33.8 ± 8.4 years old. Six patients were male, and three of them were female. HPT score decreased to 6.0 ± 1.6 after the first PRP application (*p* = 0.007) and decreased to 1.2 ± 0.8 after the last PRP session (*p* = 0.008). Five of the patients described the treatment as "very effective" after treatment with HGQ. They concluded that accelerated HL associated with COVID-19 continues in the long term and PRP treatment provides a satisfactory solution [25]. Benjamin Talei et al. [26], reported that PRP-hybridized adipose transplant hair was shown in 3 cases (including a female who suffered non-scarring alopecia following COVID-19 hospitalization and intensive care) to improve both the quality and density of hair. This treatment improved the density of hair in all patients and was characterized first by a short period of transient HL followed by new hair growth which develops starting at 4 weeks and was readily apparent at the 12-week follow-up. Results were maintained at 6-month and 1-year follow-ups [26].

### 3.4 Outcomes and endpoints

Regarding the primary outcomes, 84% of the studies analyzed (10/12) showed a direct and clear correlation between HL, TE, and COVID-19) [[15], [16], [17], [18], [19], [20], [21],[24], [25], [26]]. Regarding the secondary outcomes, 68% of the articles (4/6) displayed a positive impact of regenerative strategies against HL COVID-19 related [21,[24], [25], [26]].

### 3.5 Side effects

No major side effects have been displayed in the analyzed papers.

### 3.6 Limitations and critical assessment of study design

Performing a deep analysis of the selected studies during this investigation, a lack of standardized and widely share protocol for the procedure AD-MSCs, HFSCs, and PRP has been highlighted, as well as standardized evaluation procedures. There is a lack of shared consensus on the procedures to use. Additionally, difficulty in clearly interpreting results must be highlighted. Finally, a small size of papers (only 12) has been analyzed.

## 4 Discussion

Hypoxia in COVID-19 patients leaves detrimental effects on hair growth and hair cycling, which could justify the clinical application of PRP and stem cell-based therapies with protective effects against ischemic injury, including HFSCs, and AD-MSCs and/or Micro-needling (MND) and/or Low-Level Led Therapy (LLLT). All these therapies, and the PRP, aim to improve scalp angiogenesis. Angiogenesis involves the stimulation of endothelial cells by pro-angiogenic signals, such as vascular endothelial growth factor (VEGF), which is prevalently released by the PRP. Promoting angiogenesis and protecting the cells from ischemia are regarded as important action mechanisms in treating COVID-19-induced HL. As known, PRP has been previously used in AGA patients, and additionally, interesting results related to the use of HFSCs in these patients have been reported [27]. The PRP technique may represent a valid regenerative strategy to improve hair re-growth thanks to its capacity to release several growth factors (GFs) [[28], [29], [30]], promoting the survival of dermal papilla cells during the hair cycle via the Bcl-2 protein's activation (antiapoptotic regulator) and Akt signaling. On the other hand, current treatments for MPHL and FPHL approved by the United States (US) Federal Drug Administration (FDA) are oral Finasteride® and topical Minoxidil® in various forms, including solution and foam [30]. Since its inception, COVID-19 has imposed a pervasive, notorious impact on the public that reached even beyond the frontiers of the infection itself: psychological stress and anxiety. As reported by the World Health Organization (WHO), there has been a spiky elevation in the rates of stress and anxiety worldwide. Mandated life changes and economic insecurity as well as the fear of the unknown are important factors that contributed to the psychological burden of COVID-19. The self-isolation and quarantine measures that affect people's usual activities are expected to increase the incidence of depression, anxiety, substance (alcohol and drug) abuse, and suicide [31]. All infected patients, healthcare providers, and the public are at increased risk of psychiatric impediments during a global pandemic. Cross-sectional studies reported that 1 in every 4–5 clinicians and one in every three COVID-19 patients suffer from anxiety or mood symptoms. Early research also signified that clinically significant symptoms of anxiety, depression, distress, and post-traumatic stress disorder were present in up to one-third of the general adult population from January to April 2020 [32]. Psychological stress is a known etiology of a group of “stress-sensitive” skin conditions, including acute and chronic TE [33,34]. The physio-pathological impact of stress depends on its type and timing. Three types of psychological stress have been defined: 1- Positive stress, which is moderate, brief, and an inevitable part of normal life; 2- Tolerable stress which is more intense but occurs infrequently and gives the brain time to recover; 3- Toxic stress, which is strong in magnitude and induces prolonged activation of systemic stress responses including the sympathetic adrenomedullary system. In subjects under psychological stress, perifollicular inflammation expressed by clustering of activated macrophages and degranulation of mast cells were noticed [34]. Extended exposure to toxic levels of stress can chronically raise the circulating level of cortisol and catecholamines, which in turn underlie a wide range of disorders such as depression, anxiety, hypertension, autoimmune diseases, and cancer [35]. On the scalp, stress is shown *in-vivo* to strongly promote premature catagen and intrafollicular apoptosis in hair follicles. TE associated with COVID-19 is likely to be of an ‘immediate anagen release’ nature with massive loss of hairs due to multiple anagen-terminating signals provoked by the infection [36]. Increased levels of cortisol and catecholamines are reported to alter the hair gross cycle by affecting follicular stem cells and dysregulating the metabolism of follicular proteoglycans [37,38]. In the same way, it appears necessary to specify, that psychological stress is present, with different degrees, during the hospitalization, independently of the pathology. For this reason, the cause of HL/TE may not be specific to COVID-19, but simply due to being sick or the stress of being hospitalized.

In a recent study (published after April 1, 2022 – and for this reason not included in the analysis), the preliminary effectiveness of MND with LLLT and GFs use has been demonstrated in mild-to-moderate HL and TE related to COVID-19. In this research, the trichograms showed encouraging results with a hair density increase of 11 ± 2 hairs/cm^2^ at T1 after 20 weeks (20 weeks vs. 0 weeks) compared with baseline (58 ± 2 hairs/cm^2^ at T1 versus 47 ± 2 hairs/cm^2^ at baseline) with a not quite statistically significant difference in HR-G (*p* = 0.0690) [39]. In AGA patients (COVID-19 free) treated with MND with LLLT and GFs, an hair density increase of 81 ± 5 hairs/cm^2^ and 57 ± 7 hairs/cm^2^ respectively at T1 (12 weeks) and T2 (23 weeks) compared with baseline (173 ± 5 hairs/cm^2^ at T1 and 149 ± 9 hairs/cm^2^ at T2 versus 92 ± 2 hairs/cm^2^ at baseline) were observed using trichograms [40]. A similar result, in AGA patients, with an increase of 65 ± 5 hairs/cm^2^ in terms of hair density vs baseline has been again reported by Gentile et al. [41] confirming the results previously obtained from the use of not-activated PRP. Based on the analyzed data, the effect of the regenerative strategies has been documented both for AGA and, also for HL/TE-COVID-19 related.

## 5 Conclusions

Collected data confirmed both an increased incidence of HL and TE in COVID-19 patients, where hypoxia and reduced anagenic expression of proteoglycans have represented a potential mediating mechanism that connected HL to COVID-19. 68% of the analyzed articles (4/6) displayed a positive impact of regenerative strategies against HL COVID-19 related. Given the presence of only 6 studies (PRP n = 3; Stem Cells n = 3), of which only one reported a clear application of stem cell-based procedures, further research is needed to define standardized protocols, and large-scale PRP and regenerative therapies trials based on AD-MSCs and HFSCs still need to be conducted to confirm their effectiveness.

## Disclosures

The authors declared no potential conflicts of interest with respect to the research, authorship, and publication of this article.

## Funding

This article is part of a research project approved and supported by the University of Rome “Tor Vergata” called “*Evaluation of the potential use of regenerative strategies (Platelet Rich Plasma and Adipose-derived Mesenchymal Stem Cells) in the treatment of diseases associated with COVID-19 (Alopecia and cutaneous and subcutaneous deficiency)*” presented by the author Pietro Gentile as Principal Investigator (PI) and, approved by the Surgical Science Department of the University of Rome “Tor Vergata”, Italy with Unique Project Code (CUP): **E83C22001960005**.

## Author contributions

P.G. designed the studies, performed the procedures, analyzed the results, wrote the paper, edited the review, addressed the methodology and validation, performed the data analysis, and conducted the study as the leader; conceptualization, P.G., and S.G.; methodology, P.G., and S.G.; software, S.G.; validation, P.G., and S.G.; formal analysis, P.G.; investigation, P.G.; resources, P.G. and S.G.; data and editing curation, P.G., and S.G.; writing—original draft preparation, P.G.; writing—review and English editing, P.G. and S.G.; visualization, P.G.; supervision, P.G., and S.G.; project administration, P.G., and S.G.; funding acquisition, P.G. All authors have read and agreed to the published version of the manuscript.

## Ethical approval

Not required.

## Declaration of competing interest

The authors declare that they have no competing interests related to this study.

The corresponding author confirm to have not competing interest to this study.
